# Winter torpor expression varies in four bat species with differential susceptibility to white-nose syndrome

**DOI:** 10.1038/s41598-022-09692-x

**Published:** 2022-04-05

**Authors:** Reilly T. Jackson, Emma V. Willcox, Riley F. Bernard

**Affiliations:** 1 Department of Biological Sciences, University of Arkansas, Fayetteville, AR 72701 USA; 2 Department of Forestry, Wildlife and Fisheries, University of Tennessee, Knoxville, TN 37996 USA; 3 Department of Zoology and Physiology, University of Wyoming, Laramie, WY 82071 USA

**Keywords:** Behavioural ecology, Conservation biology, Ecological epidemiology, Ecophysiology

## Abstract

Studies examining the overwintering behaviors of North American hibernating bats are limited to a handful of species. We deployed temperature-sensitive transmitters on four species of bat that exhibit differences in their susceptibility to white nose syndrome (WNS; *Myotis grisescens*, *M. leibii*, *M. sodalis,* and *Perimyotis subflavus*) to determine if these differences are correlated with behavior exhibited during hibernation (i.e., torpor expression and arousal frequency). Mean torpor skin temperature (T_sk_) and torpor bout duration varied significantly among species (*P* ≤ 0.024), but arousal T_sk_ and duration did not (*P* ≥ 0.057). One of the species with low susceptibility to WNS, *M. leibii*, had significantly shorter torpor bout durations (37.67 ± 26.89 h) than *M. sodalis* (260.67 ± 41.33 h), the species with medium susceptibility to WNS. *Myotis leibii* also had significantly higher torpor T_sk_ (18.57 °C ± 0.20) than *M. grisescens* (13.33 °C ± 0.60), a second species with low WNS susceptibility. The high susceptibility species, *Perimyotis subflavus,* exhibited low torpor T_sk_ (14.42 °C ± 0.36) but short torpor bouts (72.36 ± 32.16 h). We demonstrate that the four cavernicolous species examined exhibit a wide range in torpid skin temperature and torpor bout duration. Information from this study may improve WNS management in multispecies hibernacula or individual species management by providing insight into how some species may differ in their techniques for overwinter survival.

## Introduction

Many bat species in North America hibernate during winter to avoid seasonal climatic changes and periods of decreased prey availability^1,2^. Hibernation is characterized by a significant drop in body temperature (T_b_) and metabolic processes that typically last several days and is sporadically punctuated by arousals to euthermic T_b_^2–4^. To survive hibernation, bats must delicately balance an energy budget that is dependent on an individual’s metabolic rate, as well as the frequency and duration of torpor and arousal bouts^5–7^. Research on hibernation behavior in North American bats has focused on several species that are either common and easily accessible or endangered^8–12^. While studies that examine hibernation behavior in numerous cave-roosting species have been conducted, comparisons across multiple species in the wild are rare^13^. Further, research into hibernation behavior across a wide range of cavernicolous species in their natural environments is warranted due to the lack of baseline information on many species and persistent risk of anthropogenic and natural disturbances^14^.

One of the most detrimental disturbances affecting cavernicolous bats in North America is the disease white-nose syndrome (WNS). Since its discovery in 2006, this disease, caused by the fungal pathogen *Pseudogymnoascus destructans* (*Pd*), has led to population declines in numerous North American cave hibernating bat species^15,16^. The fungus erodes the epidermal tissue of hibernating bats, creating lesions on the skin, muzzle, forearms, and wing membranes that lead to the disruption of homeostatic processes, and increased morbidity and mortality among infected individuals^17,18^. Marked differences in *Pd* loads and prevalence have demonstrated that susceptibility to WNS varies among species^15,19,20^. Researchers have studied a number of mechanisms that may explain host variation in susceptibility, such as microclimate preference, clustering behavior, skin microbiome, and winter activity^10,20–24^. However, the true mechanisms underlying susceptibility and resistance and/or tolerance to the disease are still not fully understood^21,22^. Understanding the reasons for heterogeneity in species susceptibility to WNS can improve management outcomes, such as maximizing host persistence and minimizing *Pd* transmission^25^.

Periodic arousal from hibernation has been hypothesized as one mechanism that may influence a species’ susceptibility to WNS^10,23,24,26^. Although repeated arousals from torpor can be detrimental to individuals infected by *Pd*, occasional arousals in regions where winters are less severe may enable populations of some species to persist^10,23,27^. Episodic activity during winter raises body temperature, often above the optimal growth range of the fungus (~ 12.5–15.8 °C)*,* and increases immune function, potentially inciting immune defense against *Pd*^28–32^. Additionally, repeated arousals throughout hibernation could allow bats to groom more frequently, thus removing *Pd* spores and reducing fungal loads^33^. Other activities such as drinking and foraging, may enable individuals to minimize some of the consequences of active WNS infection, like dehydration and starvation, by enabling individuals to supplement as needed^26,31^.

Understanding the nuances and patterns of hibernation behaviors is paramount in developing effective management recommendations targeting bat conservation. As WNS-induced mortality during hibernation can be significant, targeted intervention to minimize the effects of WNS may increase rates of survival for WNS-infected bats^5,6,16^. Data regarding the winter activity of bats may minimize the gap in knowledge regarding life history characteristics that influence susceptibility to WNS, and directly affect overwinter survival^18,31^. Therefore, we explored how hibernation behavior (i.e. torpor bout duration, arousal frequency and torpor and arousal skin temperature (T_sk_)) varies among four species with differing susceptibility to this disease: *Myotis grisescens, M. leibii, M. sodalis,* and *Perimyotis subflavus*. We selected these four species because of their perceived differences in susceptibility to WNS. Our objectives were to investigate the frequency, duration and T_sk_ of torpor and arousal bouts for individuals in a natural cave environment. We predicted that species exhibiting medium–high susceptibility to WNS would maintain torpor T_sk_ at or near the optimal growth temperature of *Pd* and short torpor bouts punctuated by frequent arousals, as an artefact of WNS infection. Conversely, we predicted that species with low susceptibility to WNS would maintain torpor T_sk_ outside the optimal growth range of *Pd* and exhibit occasional arousals from torpor, as part of their natural life history strategies.

## Methods

We conducted our study at the entrances of four cave hibernacula in east Tennessee, United States (U.S.), during three hibernation periods (November 1–March 31) of 2016–2019. Cave 1 is in Blount County in the Great Smoky Mountains National Park (GRSM) and is managed by the National Park Service (NPS). Prior to WNS, this cave was the largest known *M. sodalis* hibernaculum in the state with an estimated population of 9500 individuals^34^. White-nose-syndrome was detected in this colony during the hibernation season of 2009/10 and *M. sodalis* numbers have since declined to ~ 750 individuals^34^. *Myotis leibii* and *P. subflavus* are also encountered at this cave. Cave 2 is in Hawkins County and is managed by the Tennessee Wildlife Resources Agency (TWRA). It is one of the largest *M. grisescens* hibernacula in the state, with an estimated population of ~ 350,000 as of January 2019^34^. This cave also contains a small hibernating population of *M. sodalis* and low numbers of a few other bat species. Cave 3 is in White County and is managed by TWRA. It contains approximately 400 bats, including ~ 30 M*. sodalis* and numerous *P. subflavus*^34^. Cave 4 is also in White County and is managed by TWRA. This cave is a winter hibernaculum for 100 + *M. sodalis* and several hundred *M. grisescens*^34^. Our study includes *M. leibii* from Cave 1, *M. grisescens* from Cave 2, *M. sodalis* from Caves 2 and 4, and *P. subflavus* from Caves 1 and 3.

### Definitions

*Perimyotis subflavus* were classified as having high susceptibility to WNS because in natural settings they exhibit average fungal loads of ≥ 10^–3^ log_10_ nanograms (ng) during winter and prevalence rates ≥ 80%^15,20,35^. *Myotis sodalis* were considered medium–high susceptibility because they carry average fungal loads of 10^–3^–10^–4^ log_10_ ng and show prevalence levels of only 40–80%. Lastly, *M. grisescens* and *M. leibii* were classified as exhibiting low susceptibility to WNS because average winter fungal loads on these species are < 10^–4^ log_10_ ng and prevalence rates are < 40%^19,20,35,36^.

### Capture methods

We captured bats emerging from each cave up to four times a month using mist nets (Avinet Inc., Dryden, NY, U.S.; mesh diameter: 75/2, 2.6 m high, 4 shelves, 4–9 m wide) or harp traps (Austbat, Bat Conservation and Management Inc., Carlisle, PA, USA). We deployed mist nets and harp traps 30 min before civil sunset on nights with no rain and when temperatures were above 0 °C and left them open for up to 5 h or until temperatures fell below 0 °C. After capture, we placed individual bats into separate paper bags and held them for up to 30 min in an insulated cooler with four hand-warmers (HotHands, Dalton, GA, USA). For all individuals captured, we recorded species, sex, reproductive condition, forearm length (mm), and weight (g). Prior to release, we banded all cave hibernating species with a unique 2.4 mm or 2.9 mm numbered, lipped, alloy forearm band (Porzana, Ltd., Icklesham, East Sussex, UK).

To determine torpor and arousal bout duration and frequency, we attached a 0.27 g or 0.32 g temperature-sensitive VHF radio transmitter (LB-2X, Holohil Systems Ltd., Isanti, Ontario, Canada) to individuals of our focal bat species. We attached transmitters in the interscapular region, away from concentrations of brown adipose tissue, using surgical adhesive^8^ (Perma-Type, Plainville, CT, USA). Transmitter weight did not exceed 5–7% of an individual’s body weight^37^. In 2016/17 and 2018/19, we also entered each cave to assist TWRA and NPS with their biannual endangered species surveys, and applied radio transmitters to our focal species.

Capture, handling, sample collection, and radio-transmitter application methods were approved by the University of Tennessee Institutional Animal Care and Use Committee (IACUC 2253-0317), the American Society of Mammalogists^38^ and authorized under scientific collection permits from the U.S. Fish and Wildlife Service (TE35313B-3), NPS (GRSM-2018-SCI-1253), and TWRA (3742).

### Torpor and arousal bout data collection

We used data-logging radio-telemetry receivers (R4500SD, Advanced Telemetry Systems, Inc., Isanti, MN, USA) and dipole omnidirectional antennas (Model 13861, Advanced Telemetry Systems, Inc., Isanti, MN, USA) to record the radio signal (i.e., pulse rate) of each transmitter. Depending on the size and configuration of our cave sites, we deployed up to three antennae inside each cave, with an additional antenna stationed outside to provide radio signal detection if tagged individuals emerged. We positioned radio receivers and external batteries outside each cave to minimize disturbance of hibernating bats when downloading data. We collected data from each receiver weekly, when possible, and at a minimum biweekly.

The manufacturer calibrated each transmitter and provided a unique polynomial equation that we used to convert transmitter pulse rate to bat T_sk_. We used T_sk_ as a proxy for T_b_ to determine periods of torpor and arousal^39^. We used a conservative estimate of torpor as T_sk_ < 22 °C and arousal as T_sk_ > 22°C^40,41^. We defined arousal bouts as the period when T_sk_ > 22 °C, however individuals that gradually increased T_sk_ but did not rise to arousal T_sk_ (> 22 °C) were not included in arousal bout calculations^42^. We defined torpor bout duration as the period between two arousal bouts when T_sk_, < 22 °C^42^. Both torpor and arousal bout duration were calculated to the nearest minute.

We calculated the mean T_sk_ during each torpor and arousal bout. A torpor bout index, i.e., the number of torpor bouts divided by the number of days active, was calculated to compare among species and control for variation in transmitter lifespan. We used the same method to calculate an arousal frequency index, i.e., the number of arousals per day^43^. We characterized activity during arousals into five categories: (1) departed cave indefinitely, (2) departed cave and returned, (3) moved location in cave, (4) no movement, or (5) arrival/transmitter attachment. The activity categories were based on movement (or lack thereof) of the transmitter signal between antenna (i.e., detection of signal by different antenna) placed within and outside caves. “No movement” was defined as a transmitter that was consistently picked up on the same antenna with no gap in data collection before, during, and after an arousal bout. “Departed cave” indicated that the transmitter signal was recorded on antenna outside the cave followed by the loss of signal. “Arrival/transmitter attachment” signified when a transmitter was deployed on an individual or was detected by an antenna at a site upon reentry into the cave.

Due to small sample size, our analyses were limited to exploring differences in hibernation behavior among species only. All individuals in our study were captured at caves during hibernation, therefore natural infection of *Pd* was possible. However, due to weather limitations and the low rate of bat activity during hibernation, we were unable to recapture individuals during the life of the transmitter to determine *Pd* status. All data analyses were conducted in R^44^. We used linear mixed-effects models using the lme4 package^45^ to compare torpor bout duration, arousal bout duration, torpor temperature, and arousal temperature among species, with individual bat as a random effect. We included individuals as a random effect to correct for variation within individuals, as most bats had multiple torpor bouts and arousal events within the life of a transmitter^43,46^. For torpor bout and arousal frequency index, we used linear models with species as our explanatory variable. We log transformed all index data prior to analysis to meet assumptions of normality and homogeneity of variance^47,48^. Post-hoc tests were conducted using least square means using the emmeans package^49^.

## Results

We deployed 77 temperature-sensitive radio transmitters and successfully recorded T_sk_ data from 22 individuals (28.6% of bats tagged). After initial transmitter attachment, the transmitter signals of 40 individuals (51.9% of tagged bats) were never detected on cave antenna during the life of their transmitters (15–21 days). Fifteen individuals (19.5% of bats tagged) were detected by cave antennas after the night of transmitter attachment but did not remain in antennae range long enough for data collection.

### Mean torpor and arousal temperature

**Figure 1:**
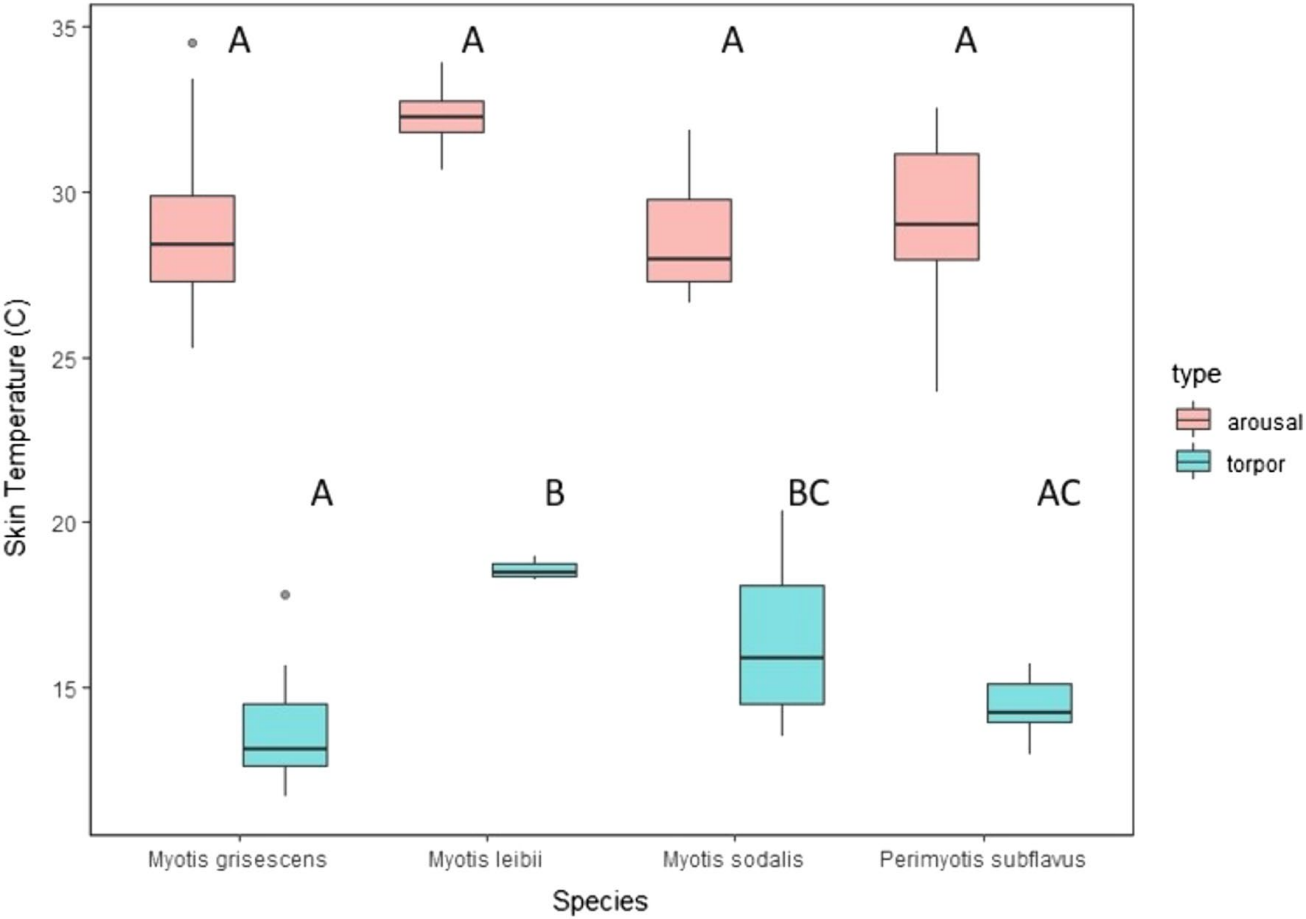
Mean skin temperature (℃; T_sk_) during torpor and arousal of four bat species tracked with temperature-sensitive radio transmitters at four cave hibernacula in Tennessee during hibernation (November 1–March 31) of 2016–2019. Dots above plots represent outlying data. *Boxplot data followed by the same uppercase letter not significantly different (*P* > 0.05).

There was considerable variation in the mean T_sk_ during torpor and arousal of the four species studied (Table 1). There was an effect of species on mean torpor T_sk_ (*P* = 0.004; Fig. 1). *Myotis leibii* had the highest mean torpor T_sk_ (18.57 ± 0.20 °C), whereas *M. grisescens* had the lowest mean torpor T_sk_ (13.72 ± 0.60 °C). Post-hoc tests indicated that *M. leibii* had a higher mean torpor T_sk_ than *P. subflavus* (14.44 ± 0.36 °C; *P* = 0.021), and *M. grisescens* had a lower mean torpor T_sk_ compared to *M. leibii* (*P* = 0.005) and *M. sodalis* (16.48 ± 0.79 °C; *P* = 0.019).

**Table 1:** The number of transmitters detected, mean skin temperature (T_sk_) during torpor and arousal, and mean torpor (hours) and arousal (minutes) bout durations of four bat species tracked with temperature-sensitive radio transmitters at four cave hibernacula in Tennessee during hibernation (November 1–March 31) of 2016–2019.

Species	Number of transmitters detected (no.)	Torpor T_sk_ (°C; $\overline{x }$ ± SE)	Arousal T_sk_ (°C; $\overline{x }$ ± SE)	Torpor bout duration (h; $\overline{x }$ ± SE)^a^	Arousal bout duration (min; $\overline{x }$ ± SE) ^a^
*Myotis grisescens*	8	13.72 ± 0.60 _A_	29.01 ± 0.64 _A_	162.13 ± 48.79_AB_	84.88 ± 15.08 _A_
*Myotis leibii*	2	18.57 ± 0.20 _B_	32.29 ± 0.67 _A_	37.67 ± 26.89_A_	103.75 ± 60.62 _A_
*Myotis sodalis*	9	16.48 ± 0.79 _BC_	28.59 ± 0.38 _A_	260.67 ± 41.33_B_	66.94 ± 6.82 _A_
*Perimyotis subflavus*	3	14.44 ± 0.36 _AC_	28.99 ± 0.84 _A_	72.36 ± 32.16_A_	79.27 ± 21.04 _A_

^a^$\overline{x }$ ± SE in the same column followed by the same uppercase letter are not significantly different (*P* > 0.05).

*Myotis leibii* had the highest mean arousal T_sk_ (32.29 ± 0.67 °C) and *M. sodalis* had the lowest mean arousal T_sk_ (28.59 ± 0.38 °C; Table 1; Fig. 1). However, there was no effect of species on mean arousal T_sk_ (*P* = 0.057).

### Mean torpor and arousal bout duration

**Figure 2:**
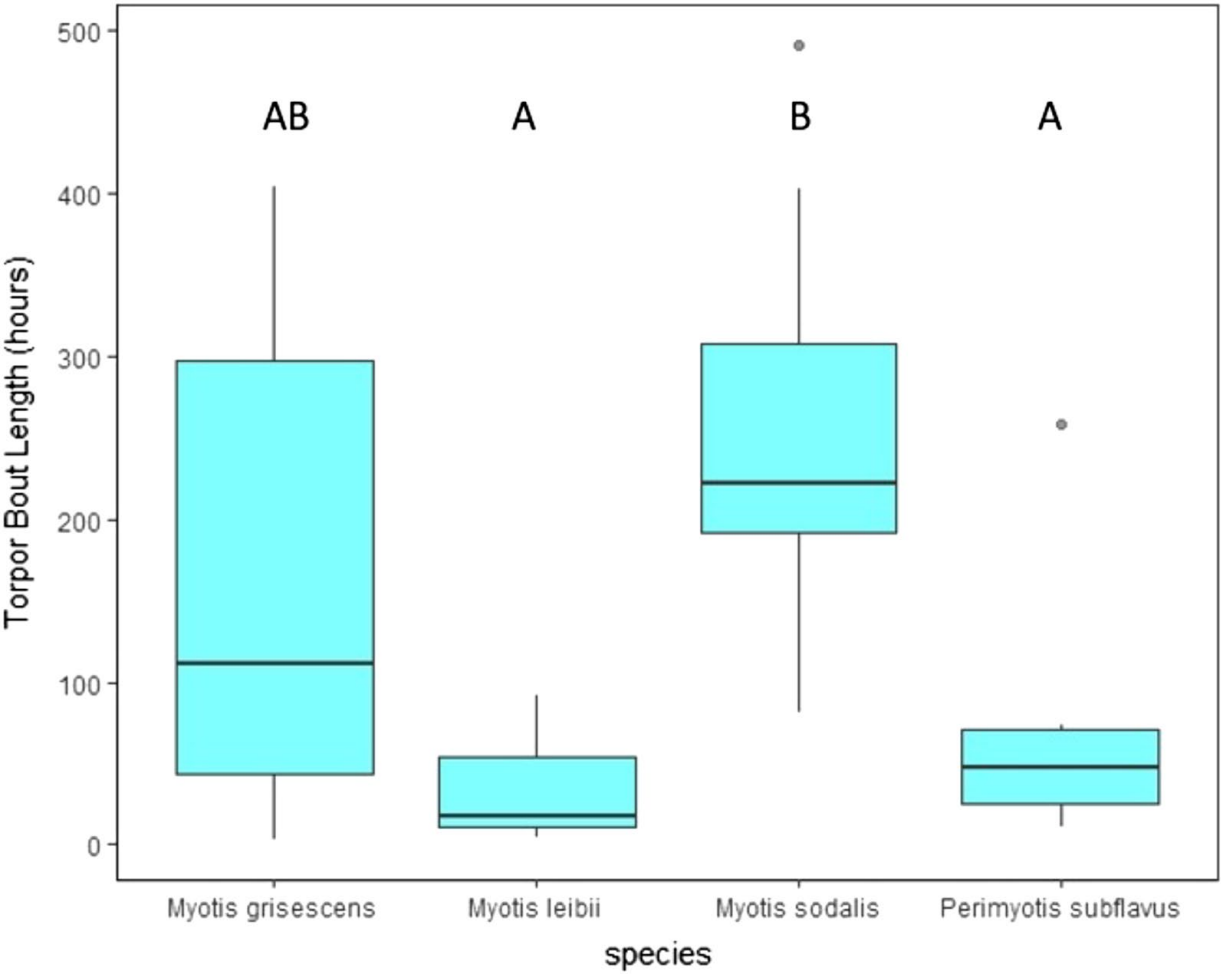
Mean torpor bout duration (hours) of four bat species tracked with temperature-sensitive radio transmitters at four cave hibernacula in Tennessee during hibernation (November 1–March 31) of 2016–2019. Dots above plots represent outlying data. *Boxplot data followed by the same uppercase letter not significantly different (*P* > 0.05).

**Figure 3:**
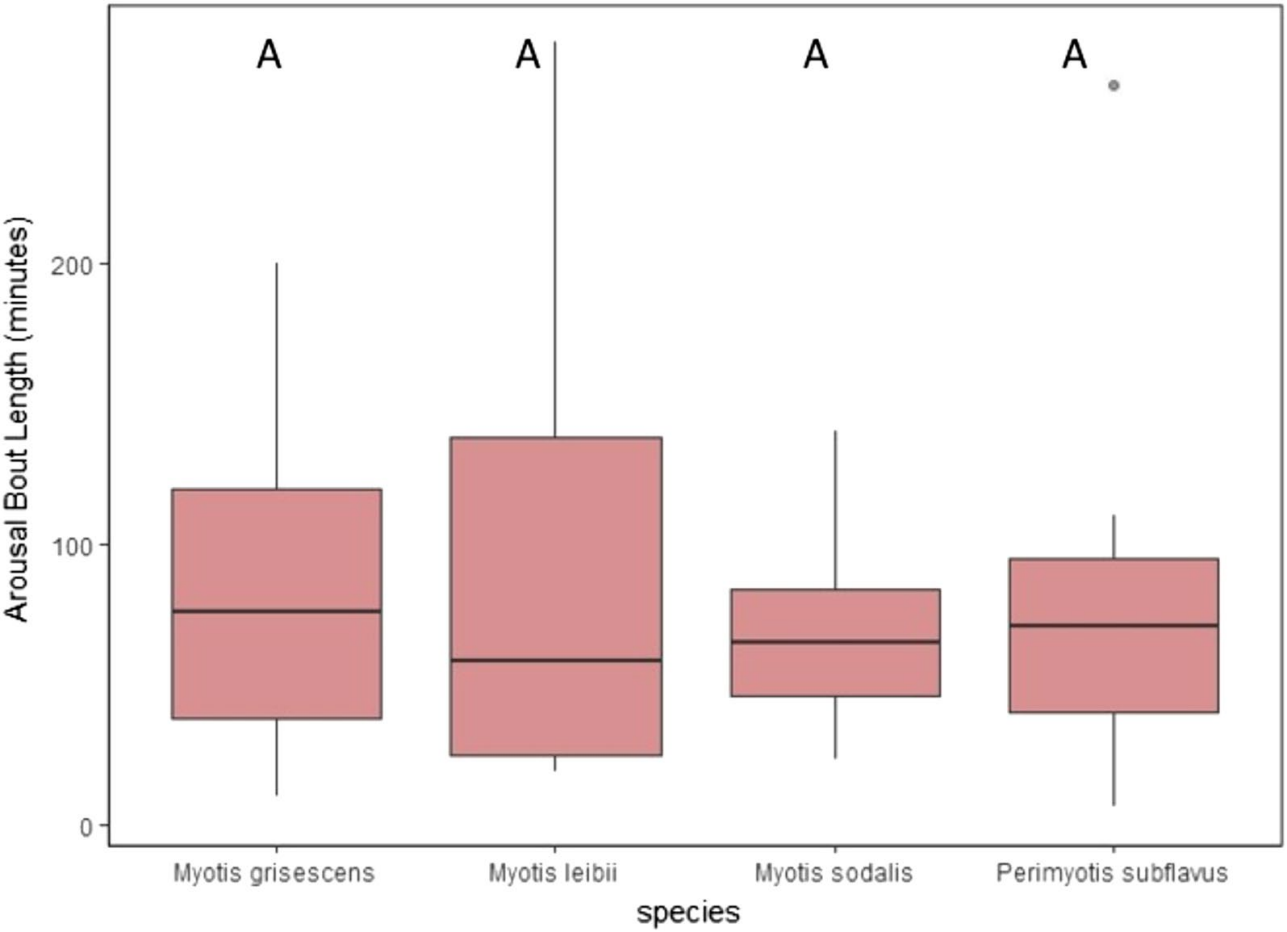
Mean arousal bout duration (min) of four bat species tracked with temperature-sensitive radio transmitters at four cave hibernacula in Tennessee during hibernation (November 1–March 31) of 2016–2019. Dots above plots represent outlying data. *Boxplot data followed by the same uppercase letter not significantly different (*P* > 0.05).

Mean torpor bout duration across all species was less than 11 days (Table 1; Fig. 2). There was an effect of species on mean torpor bout duration (*P* = 0.024). *Myotis sodalis* had a significantly longer mean torpor bout duration (260.67 ± 41.33 h) than *P. subflavus* (72.36 ± 32.16 h; *P* = 0.029) and *M. leibii* (37.67 ± 26.89 h; *P* = 0.029). *Myotis leibii* had the longest mean arousal bout duration (103.75 ± 60.62 min), whereas *M. sodalis* had the shortest mean arousal bout duration (66.94 ± 6.82 min; Table 1; Fig. 3). However, there was no effect of species on mean arousal bout duration (*P* = 0.655).

### Activity throughout hibernation

**Figure 4:**
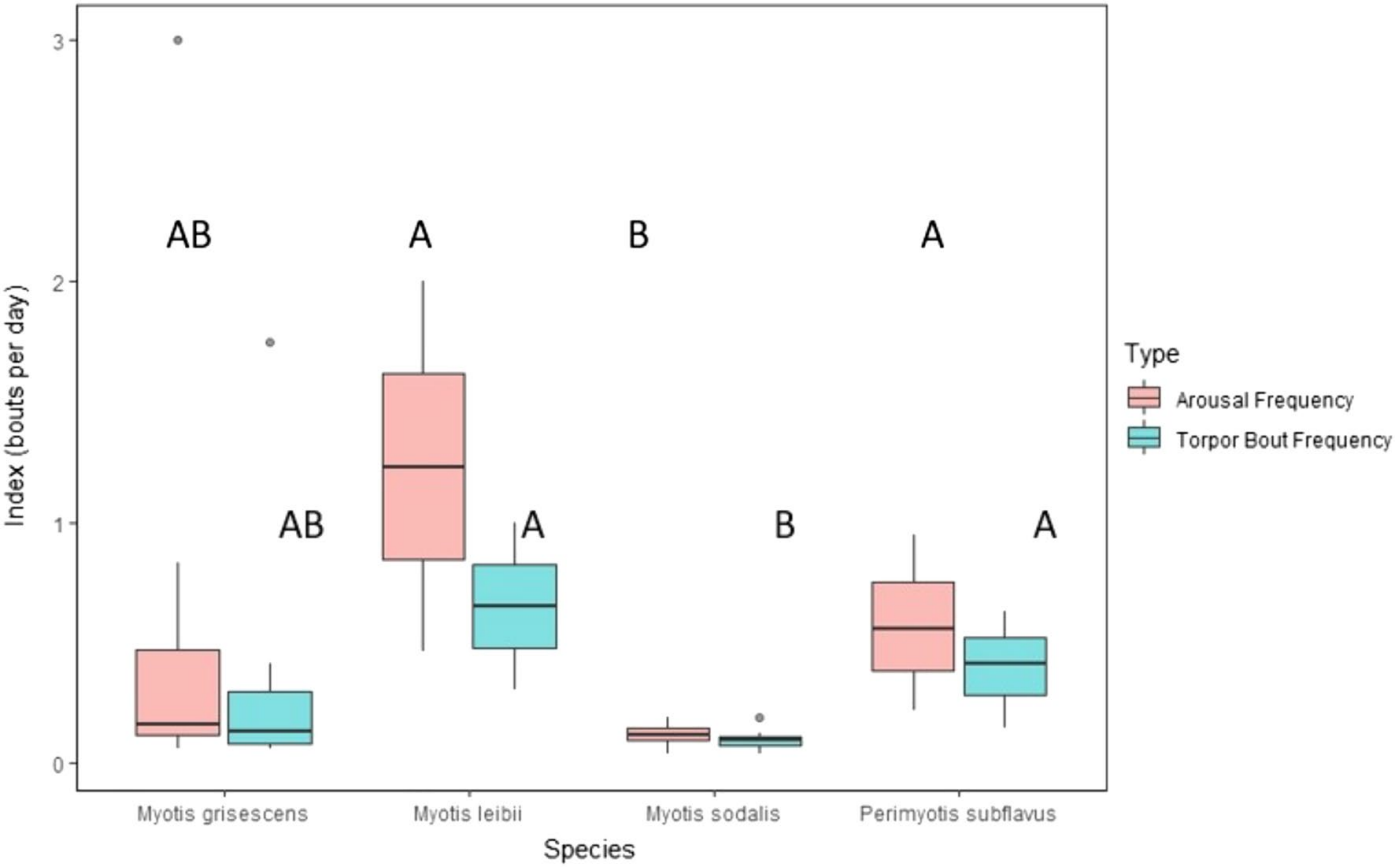
Torpor bout and arousal bout frequency indices (bouts/day) of four target bat species tracked with temperature-sensitive radio transmitters at four cave hibernacula in Tennessee during hibernation (November 1–March 31) of 2016–2019. Dots above plots represent outlying data. *Boxplot data followed by the same uppercase letter not significantly different (*P* > 0.05).

The number of torpor and arousal bouts per species during hibernation varied significantly across species (Fig. 4). *Myotis leibii* had the highest torpor bout (0.654 ± 0.346 torpor bouts/day) and arousal frequency indices (1.231 ± 0.769 arousals/day), whereas *M. sodalis* had the lowest indices (torpor bout index: 0.097 ± 0.015; arousal frequency index: 0.117 ± 0.016). There was an effect of species on both torpor bout index (*P* = 0.026) and arousal frequency index (*P* = 0.023). Post hoc tests indicate *M. leibii* and *P. subflavus* had a greater torpor bout index and arousal frequency index than *M. sodalis* (*P* ≤ 0.028). However, the torpor bout index and arousal bout index of *M. leibii* and *P. subflavus* were similar to that of *M. grisescens* (*P* ≥ 0.087).

Activity during arousals varied, with 34.69% of arousals resulting in bats moving out of range of the antennas and returning within five hours (n = 17/49 arousal events). In 12.24% of arousal events, bats left the cave and were never recorded again during the life of the transmitter (n = 6/49). There was no indication of movement within or out of the cave in 20.41% of recorded arousal events (n = 10/49).

## Discussion

We found significant differences in torpor T_sk_, torpor bout duration, torpor bout index, and arousal frequency index among four bat species that exhibit variation in their susceptibility to WNS. In contrast, we found no significant difference in arousal T_sk_ or arousal bout duration among species. Although we were unable to gather data from all deployed transmitters, we were able to fill in knowledge gaps regarding the hypothesized differences in hibernation behavior across multiple bat species in a natural environment. By understanding the differences in these winter torpor and arousal characteristics in bat species with varying degrees of susceptibility to WNS, we can continue to improve our understanding of why some species experience high rates of morbidity and mortality from the disease. Given that *Pd* grows and colonizes hosts more efficiently in certain microclimates^50^ identifying T_sk_ (as a proxy for T_b_) and winter activity of bats that hibernate in natural cave environments can help elucidate factors that may increase the risk of *Pd* infection.

Prior to our study, we expected *P. subflavus* and *M. sodalis*, the species with medium–high susceptibility to WNS, to maintain torpor T_sk_ at or near the optimal growing temperature of *Pd,* with *M. grisescens* and *M. leibii*, our species with seemingly low susceptibility, at torpid T_sk_ levels outside the optimal *Pd* growth range (~ 12.5–15.8 °C)^32^. Interestingly, *M. leibii* maintained the highest T_sk_ during both torpor and arousal and were consistently active throughout the life of the transmitter. Data from winter captures and diet analysis from this region indicate *M. leibii* arouse from torpor regularly and engage in foraging throughout the hibernation period, frequently leaving hibernacula on winter nights, when temperatures are greater than − 1 ℃^20,27^. This species is known to occupy a large portion of eastern North America, with the northern extent of its limit in southern Canada^50^. Given this species’ ability to survive severe winters throughout southern Canada and the northern US, *M. leibii* in Tennessee may be physiologically adept at handling winters in the southern US, where temperatures are comparably mild and rarely remain below freezing for extended periods^23,51^. Therefore, the frequent winter activity documented by *M. leibii* in our study may largely be influenced by the species’ inherent cold tolerance. Such tolerance may be necessary for survival in the northern portion of their range and may play an important role in the species apparent low susceptibility in the southern US. In areas where winter temperatures are rarely below freezing, *M. leibii* may be able to regularly maintain both torpor and arousal T_sk_ above the optimal growth temperature of *Pd*, which may reduce exposure to and minimize serious *Pd* infection^32^. Short torpor bouts punctuated by regular foraging activity may also help the species maintain immune system function, as well as supplemental energy reserves, allowing them to persist with low level infections without significant mortality^15,19,20,39,52,53^. Additionally, our findings are consistent with pre-WNS data from this latitude, suggesting that this activity regime is not an artifact of WNS, but an inherent life history characteristic^52,54,55^. While activity during winter does not completely explain the persistence of *M. leibii* throughout the WNS epizootic, it is possible that their rates of frequent activity are beneficial to their survival^10,20,24,26,36^.

Conversely*, P. subflavus* maintained short torpor bout durations similar to *M. leibii*, but low torpor T_sk_, akin to *M. grisescens* and within the optimal *Pd* growth range^32^. These short torpor bouts at low T_sk_ could explain some of the mortality seen in *P. subflavus* due to WNS. Compared to higher T_sk_, bats expend more energy arousing from torpor when they hibernate at low T_sk_, which is otherwise useful for maintaining long, energy-saving torpor bouts^56^. However, in our system, *P. subflavus* was arousing (on average) every 3 days from a low torpor T_sk_, which likely puts a severe and recurring drain on energy reserves and may contribute to mortality in this species^42^. Data from the region also suggests that while *P. subflavus* maintain a low level of activity during much of the hibernation period, activity increases in February, months earlier than the documented emergence from hibernation in pre-WNS literature for this longitude^57,58^. The increase in activity, which correlates with a rise in fungal loads and prevalence of *Pd* on *P. subflavus*, and associated energy drain may be a product of sickness behavior in infected *P. subflavus*^20,36,42,59^. It is possible that the characteristics of winter activity in *P. subflavus* documented during our study are WNS-induced behaviors, rather than a healthy life history strategy, and could greatly reduce the overwinter survival rate of *P. subflavus* in this area.

In contrast to *M. leibii* and *P. subflavus*, *M. grisescens* and *M. sodalis* had longer torpor bouts, with *M. grisescens* torpor T_sk_ the lowest across our focal species and *M. sodalis* torpor T_sk_ just below *M. leibii*. Given the limited similarities in hibernation behavior between these two species, aspects other than winter activity likely influence their risk of *Pd* infection and WNS manifestation. *Myotis grisescens* are year-round cave dwellers that are large-bodied and highly gregarious, hibernate in cold microclimates, and can be active throughout winter^20,60^. Dietary analysis of *M. grisescens* guano collected from active bats during winter indicates that this species is regularly eating aquatic insects through the hibernation period, likely from waterways in or near hibernacula that provide ample foraging opportunities year-round^27^. Coupled with adequate energy intake throughout winter, the physical and behavioral traits of *M. grisescens* may enable this species to resist or tolerate *Pd* infections with minimal adverse effects on survival, much like *M. leibii*^20,53^. While *M. leibii* may be resisting *Pd* infection via regular arousals and shallow torpor bouts, *M. grisescens* may tolerate *Pd* due to their preference for long torpor bouts at cold temperatures.

Alternatively, we found that *M. sodalis* maintained a relatively high torpid T_sk,_ similar to *M. leibii*, yet had significantly longer torpor bouts and less frequent arousals than seen in *M. leibii*. Previous research on the fungal load and infection intensity of WNS for *M. sodalis* indicates that hibernating populations of the species experience mid-range intensity of fungal load with relatively high prevalence^15,20,36^. Although we found the species maintains similar torpor T_sk_ as *M. leibii*, the difference in activity regimes between the two species may allow for increased fungal growth on *M. sodalis*, despite maintaining torpid T_sk_ slightly above the optimal growth temperature of *Pd*. While there are likely myriad factors influencing susceptibility, it is possible that higher T_sk_, coupled with low activity, is not enough to reduce *Pd* growth on this species^36^. More research is needed to explore the drivers of WNS susceptibility in this species, given that there are highly variable patterns in pathogen dynamics, but still a marked decline in their population^16^.

While our success at documenting the hibernation behaviors of cavernicolous bats in their natural environments was limited, our methods illuminated issues with the long-held belief that many hibernating bat species practice strict roost fidelity during winter^55,61,62^. Over 50% of tagged bats did not stay within range of the cave antenna that would have allowed for the collection of temperature data. Specifically, several *M. grisescens* and *M. leibii* were recorded leaving the hibernacula via aerial radiotelemetry and either roosting on the landscape and never returning to the cave during the life of the transmitter or disappearing completely, several kilometers away from the cave capture site. While the reason for signal disappearance is not wholly understood, aerial telemetry experts believe that bats with disappearing signals most likely moved to other subterranean sites, outside the range of the antenna (S. Samoray, pers. comm.). Although roost fidelity is known to fluctuate based on availability of roosts, hibernacula fidelity has long been either unknown or assumed to be high^55,61,63^. Our results provide definitive proof that hibernacula fidelity largely varies among individuals, especially for species like *M. grisescens* and *M. leibii*. Many agencies may base proposed management actions on previous assumptions, possibly missing secondary hibernacula important for overwinter survival^64^. Therefore, many researchers may mistakenly assume high hibernacula fidelity in their study species and develop study methodology based on this assumption. More work is needed to understand winter roost fidelity in cavernicolous species and what it may mean for WNS management.

Due to small sample size for *M. leibii* and *P. subflavus,* our ability to analyze characteristics that may have influenced hibernation behavior within species, such as age, sex, body condition, time of year, cave microclimate, or *Pd* status is limited.However, even with these limitations, our study is the first to directly compare hibernation behavior across several species of cavernicolous bats in their natural overwintering environment. The information we collected is an important step towards understanding the intricacies of hibernation behavior of cavernicolous bats, although more work is obviously needed to identify the drivers of the differences we found. Recognizing the variations in hibernation behavior is important to both researchers and managers as strategies aimed at maintaining bat populations affected by WNS may be more effective if specifically targeted to species with the highest known susceptibility. Some low-cost actions may be to reduce anthropogenic disturbances at or near hibernacula used by *M. sodalis* and *P. subflavus*. Additionally, improving, or augmenting habitat for increased prey availability around hibernation sites may be beneficial to species that remain active on the landscape during winter, such as *M. grisescens* and *M. leibii*. Given that two of our four focal species are listed as Endangered in the U.S. (*M. grisescens* and *M. sodalis*) and one (*P. subflavus*) is petitioned for listing, any management efforts that can increase survival of individuals and persistence of populations are crucial for successful conservation of these species.

## Data availability

The datasets generated during and/or analysed during the current study are available from the corresponding author on reasonable request.
